# A Pilot Study of Orthopedic Applicants Regarding Sub-internship Number, Cost, and Preferences

**DOI:** 10.7759/cureus.87785

**Published:** 2025-07-12

**Authors:** Matthew J Schultz, Samantha Riebesell, Rex W Lutz, Joseph McCahon, Joyce D Suarez, James Purtill, Joseph Daniel

**Affiliations:** 1 Department of Orthopedic Surgery, Jefferson Health New Jersey, Stratford, USA; 2 Department of Orthopedic Surgery, Rothman Orthopaedic Institute, Philadelphia, USA; 3 Department of Orthopedic Surgery, Philadelphia College of Osteopathic Medicine, Philadelphia, USA; 4 Division of Adult Reconstruction, Rothman Orthopaedic Institute, Philadelphia, USA; 5 Department of Orthopedics, Rothman Orthopaedic Institute, Philadelphia, USA

**Keywords:** audition rotations, away rotations, medical student, orthopedic applicant, orthopedic education, orthopedic surgery sub-internships

## Abstract

Background and objective

This pilot study surveyed orthopedic applicants regarding their experience with sub-internship (sub-I) rotations (number, cost, and selection criteria), seeking to identify potential barriers, and their implications for residency selection for future study.

Methods

Applicants to two orthopedic residencies in the Philadelphia area received surveys regarding the number/cost of sub-Is, factors affecting rotation site selection, and the influence of people/experiences on the rank list. Surveys were available between the rank list certification deadline and the match week.

Results

One hundred ninety out of 936 (20.3%) applicants completed the survey. One hundred thirty-three (70%) respondents completed ≥4 rotations (inclusive of home rotations). Osteopathic applicants completed more rotations than allopathic applicants (mean {M}=5.1 versus 3.8, p<0.001). Fifty-seven (30.0%) respondents stated that their rotation time was limited by cost, with 116 (61.1%) reporting a monthly cost of >$1600. Factors that were most important to applicants when selecting a rotation site were geographic location (4.8), program reputation (3.9), and word of mouth (3.8), while factors that were less important to applicants included social media presence (1.9), scholarship opportunities (2.1), and program website (2.5). Women valued program diversity more than men (3.6 versus 2.5, p<0.001). Residents (4.8) were the most influential people when determining rank order.

Conclusions

Current orthopedic applicants may be completing more sub-Is at a greater cost. Larger, nationally representative samples should seek to confirm these findings and determine whether sub-Is represent a barrier to entry in orthopedics. Our findings also highlight applicant preferences when selecting sub-Is and determining rank order. This should help programs better direct recruitment efforts and provide a more applicant-focused rotation experience.

## Introduction

Sub-internships (sub-Is), also known as audition rotations or away rotations (when completed at an outside institution), are clinical rotations completed by fourth-year medical students in the specialty in which they intend to apply for residency. Sub-Is provide an educational experience while allowing applicants to create professional connections, obtain letters of recommendation, and demonstrate qualifications as a residency candidate [1]. A 2020 survey of US medical school graduates reported that 98.9% of graduates intending to match into orthopedics completed at least one away rotation [2]. This approach is supported by studies that report that orthopedic applicants are much more likely to receive interview offers and match at programs at which they rotated [3-5].

While valuable, sub-Is require considerable investment of time, energy, and finances. A 2015 survey of residency applicants found that the average cost of housing, travel, and transportation was $956 per rotation [6]. Some applicants may not be able to afford this expense and therefore may opt out of sub-I rotations, potentially placing them at a competitive disadvantage.

While much is known about the importance of sub-Is in the orthopedic application process, little is known about what matters to applicants while on these rotations. A better understanding of the people and activities that applicants find influential when determining their rank order list can help programs provide a more tailored rotation experience. Similarly, insight into the factors that applicants consider important when determining where to apply for sub-Is can help programs focus recruitment efforts. Finally, understanding the average number, duration, and cost of sub-Is would help to appreciate their role in the current orthopedic application process, particularly after the United States Medical Licensing Examination (USMLE) Step 1 and Comprehensive Osteopathic Medical Licensing Examination (COMLEX) Level 1 transition to pass/fail scoring. The purpose of this pilot study was to survey applicants to two affiliated orthopedic residency programs in the Philadelphia metropolitan area regarding sub-I experiences (rotation count, cost, and selection criteria), to identify potential barriers and their implications for residency selection.

## Materials and methods

Institutional review board approval was obtained prior to study implementation. The Institutional Review Board of Thomas Jefferson University issued approval iRISID-2023-1563. Anonymous surveys were distributed via Research Electronic Data Capture (REDCap) (Vanderbilt University, Nashville, TN) to all applicants to two orthopedic residencies in the Philadelphia metropolitan area. Surveys were emailed to applicants the day after the rank list certification deadline (February 29, 2024), followed by two reminder emails. The survey was closed the night before applicants were notified of their initial match status (March 10, 2024). Because of this timing, applicants were unaware of their match status, and this information was not collected.

The survey included questions regarding their experience on sub-Is (see Appendices to review survey). Questions pertained to the number and duration of rotations, housing options, cost, and whether their time on rotations was limited by cost. Reported weekly costs were converted to monthly costs by multiplying responses by four for ease of understanding and to allow more direct comparison to prior studies. Applicants also indicated the influence of certain individuals and experiences during rotations on their rank order list using a five-point sliding scale, with 1=not influential/useful and 5=extremely influential/useful. These factors were derived from a prior similar study conducted by O’Donnell et al. [7]. Finally, using factors derived from the 2023 National Resident Matching Program (NRMP) Applicant Survey, applicants indicated the importance of certain factors when determining where to apply for sub-Is using a five-point sliding scale [8]. The survey was reviewed by the authors and a cohort of medical students interested in orthopedic residency to improve face validity and content clarity.

Responses were analyzed using RStudio (version 4.1.2, R Foundation for Statistical Computing, Vienna, Austria). Descriptive statistics were performed on the entire cohort. When calculating the mean (M) number of sub-Is, applicants who completed >5 rotations were assumed to have completed six rotations. Mean importance and standard deviation (SD) were reported for the sliding scale responses. Applicants were stratified by gender and type of medical school (US allopathic, US osteopathic, or international medical graduate {IMG}), and responses were compared between groups. Chi-square tests were used for categorical and ordinal data, and t-tests or Mann-Whitney U tests were used for interval data. A Bonferroni correction was utilized, resulting in an adjusted alpha of 0.0185.

## Results

Responses were received from 190 out of 936 (20.3%) applicants. Table 1 presents demographics. The small number of IMG respondents and respondents identifying as neither men nor women precluded meaningful statistical analyses on these cohorts.

**Table 1:** Applicant Demographics IMG: international medical graduate

	N (%)
Gender	
Man	135 (71.1)
Woman	53 (27.9)
Others	2 (1.1)
Medical school	
US allopathic	128 (67.4)
US osteopathic	53 (27.9)
IMG	9 (4.74)

Table 2 presents sub-I characteristics. One hundred thirty-three (70%) respondents completed ≥4 sub-Is. The overall mean (standard deviation {SD}) number of rotations was 4.2 (1.2), and the median was 4. There was a significant difference in the number of rotations by applicant type, with osteopathic applicants completing more rotations (M=5.1; SD=1.1) than allopathic applicants (M=3.8; SD=0.9) (χ²(5, N=181)=73.43; p<0.001; Cramér’s V=0.637).

**Table 2:** Away Rotation Characteristics *Significant difference between applicant type (osteopathic versus allopathic) (p<0.001)

Number of away rotations*	N (%)
1	2 (1.1)
2	10 (5.3)
3	45 (23.7)
4	65 (34.2)
5	35 (18.4)
>5	33 (17.4)
Average duration of away rotations	
≤2 weeks	1 (0.5)
4 weeks	182 (95.8)
>4 weeks	7 (3.7)
Time needed on rotation to make a decision about rank order	
<1 week	6 (3.2)
1-2 weeks	50 (26.3)
2-4 weeks	128 (67.4)
>4 weeks	6 (3.2)

Table 3 presents information regarding rotation cost and housing. Time on sub-Is was limited secondary to cost for 57 (30%) respondents. The monthly cost was >$1600 for 116 (61.1%) respondents, with housing accounting for at least $1200 per month in 101 (53.2%). There was a difference in housing cost between applicant types (p=0.007) and overall cost between genders (p<0.001). For 124 (65.3%) respondents, housing was not offered. Osteopathic applicants were more likely than allopathic applicants to have housing offered at least sometimes (χ²(2, N=181)=36.52; p<0.001; Cramér’s V=0.449). Among respondents required to find their own housing, 79 (63.7%) utilized a housing/room rental, 25 (20.2%) lived with a friend, and 11 (8.9%) only rotated locally.

**Table 3:** Cost Characteristics for Away Rotations *Significant difference between gender (p<0.001) #Significant difference between applicant type (allopathic versus osteopathic) (p=0.007)

	N (%)
Average monthly overall cost#	
$0-400	1 (0.53%)
$401-800	14 (7.37%)
$801-1200	19 (10.0%)
$1201-1600	32 (16.8%)
$1601-2000	35 (18.4%)
$2001-4000	50 (26.3%)
>$4000	31 (16.3%)
Unsure	8 (4.21%)
Average monthly cost of housing*#	
Housing was free	16 (8.42%)
$1-400	2 (1.05%)
$401-800	17 (8.95%)
$801-1200	44 (23.2%)
$1201-1600	33 (17.4%)
$1601-2000	29 (15.3%)
>$2000	39 (20.5%)
Unsure	10 (5.26%)
Time on rotations limited by cost?	
Yes	57 (30%)
No	133 (70%)

**Figure 1:**
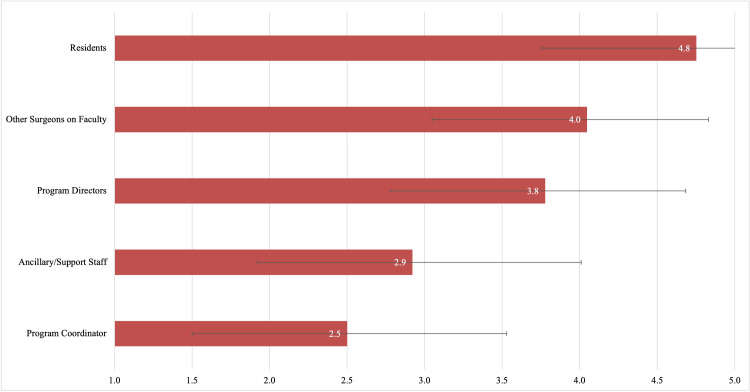
The Influence of Specific People Encountered During Away Rotations on Applicants’ Rank Order Lists Applicants responded using a five-point sliding scale where 1=not influential and 5=extremely influential. Numbers represent the mean. Error bars represent standard deviation

**Figure 2:**
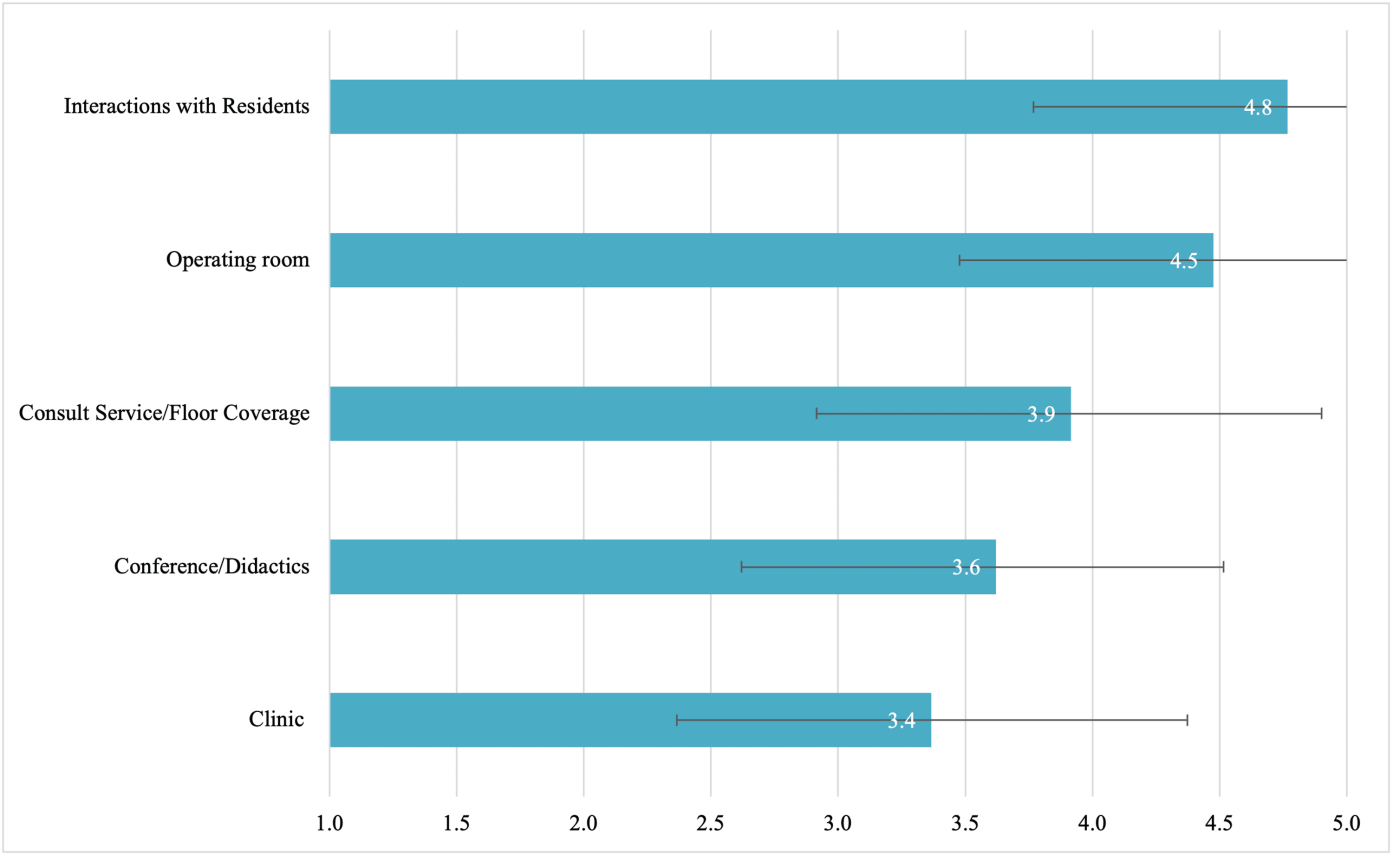
The Usefulness of Different Experiences During Away Rotation When Determining Rank Order List Applicants responded using a five-point sliding scale where 1=not influential and 5=extremely influential. Numbers represent the mean. Error bars represent standard deviation

The influence of specific people encountered during rotations when determining rank order is presented in Figure 1, sorted in descending order of importance. There were no significant differences when stratifying by gender or applicant type. The usefulness of different experiences during rotations when determining rank order is presented in Figure 2, sorted in descending order of importance. There were no significant differences when stratifying by gender or applicant type.

**Figure 3:**
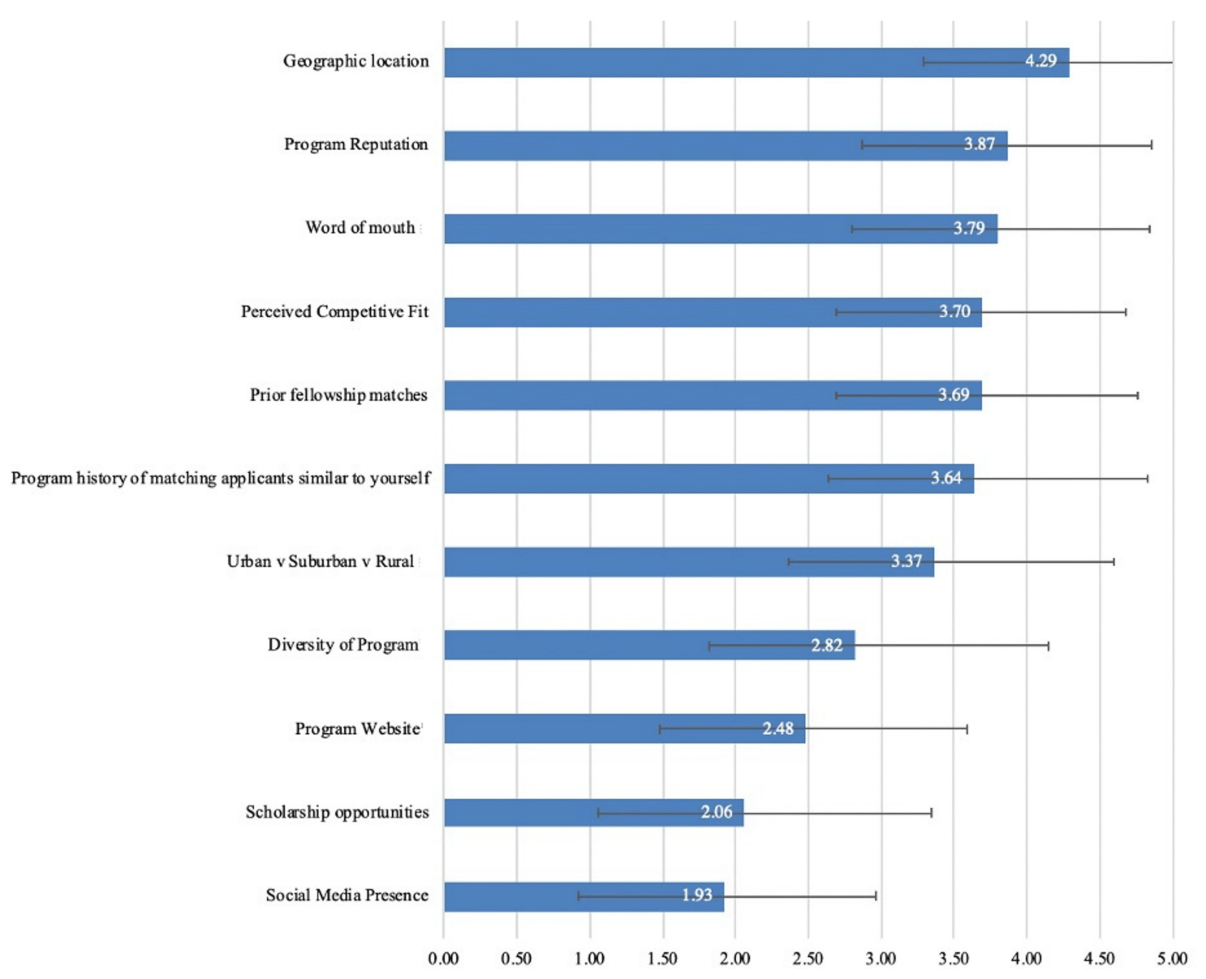
Factors Considered by Applicants When Determining Where to Apply for Away Rotations Applicants responded using a five-point sliding scale where 1=not influential and 5=extremely influential. Numbers represent the mean. Error bars represent standard deviation

**Figure 4:**
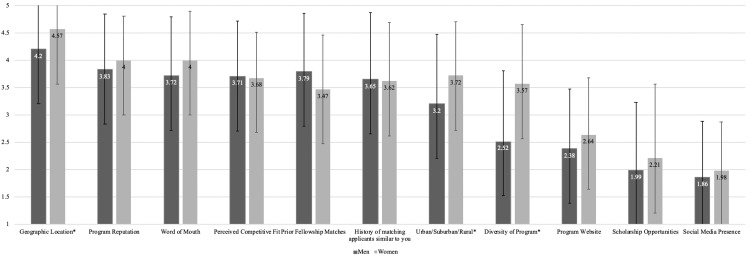
Factors Considered by Applicants When Determining Where to Apply for Away Rotations by Gender Applicants responded using a five-point Likert scale where 1=not influential and 5=extremely influential. Numbers represent the mean. Error bars represent standard deviation *Women found geographic location (p=0.008), urban/suburban/rural settings (p=0.004), and program diversity (p<0.001) more influential than men

**Figure 5:**
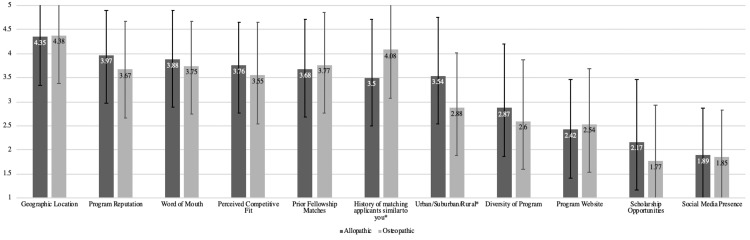
Factors Considered by Applicants When Determining Where to Apply for Rotations by Applicant Type (Allopathic Versus Osteopathic) Applicants responded using a five-point sliding scale where 1=not influential and 5=extremely influential. Numbers represent the mean. Error bars represent standard deviation *Compared to allopathic applicants, osteopathic applicants found a history of matching applicants similar to themselves more important (p=0.005) and urban/suburban/rural setting less important (p=0.003)

Factors considered when determining where to apply for sub-Is are presented in Figure 3, and Figure 4 compares these factors by gender. Women placed greater importance on program diversity (p<0.001), geographic location (p=0.008), and urban/suburban/rural program setting (p=0.004) compared with male applicants. Figure 5 compares these factors by applicant type. Osteopathic applicants placed less importance on urban/suburban/rural program setting (p=0.003) but greater importance on prior history of matching applicants similar to themselves (p=0.009) compared with allopathic applicants. Twenty-two (11.6%) respondents stated that other factors not listed in the survey impacted their decision regarding where to apply; a list of these factors is provided in the Appendices.

## Discussion

Sub-Is are an important component of the orthopedic residency application process. In a survey of program directors, 86% of respondents stated that completing an away rotation increases an applicant’s competitiveness at the program in which they rotated [7]. With the transition of USMLE Step 1 and COMLEX Level 1 to pass/fail scoring in 2022, these rotations may gain greater emphasis in the coming years. This study offers valuable insight into three key aspects of the applicant experience: the number and cost of away rotations, factors influencing the selection of rotation sites, and determinants of rank order list decisions. Notably, over a quarter of applicants reported spending an average of $2000-$4000 per rotation, highlighting the significant financial burden associated with the application process. When choosing sub-I rotations, geographic location emerged as the most influential factor, suggesting that proximity or regional preferences play a central role. Importantly, resident interactions had the greatest impact on applicants’ final rank lists, emphasizing the critical role of residents in developing relationships with applicants.

Number and cost of rotations

We found that 70% of applicants completed ≥4 sub-Is with a mean (SD) of 4.2 (1.2) and a median of 4. A survey of orthopedic applicants in the 2014-2015 match cycle reported a mean (median) number of away rotations of 2.47 (2) [7]. A 2020 NRMP survey reported a median number of rotations for applicants intending to match into orthopedics of 3 [2]. A notable limitation in the ability to directly compare our findings to prior studies is that these studies did not include home institution rotations in their count, while the terminology in our survey (“audition/sub-I rotations”) was intended to include home rotations. Future studies should consider surveying/reporting the total number of sub-I rotations alongside the number of “away rotations” (i.e., rotations completed at an outside institution). In addition to more direct comparison to prior studies, this would allow more accurate estimation of the overall cost.

Nearly half of osteopathic applicants completed at least five away rotations, whereas only 12.5% of allopathic applicants did the same. While the reasons for these observed differences in our study are not known, the orthopedic match rate for US allopathic seniors in 2024 was 72% compared to 46% for US osteopathic seniors [9]. Oftentimes, osteopathic medical schools do not have an associated orthopedic residency program and require applicants to seek out other programs. Osteopathic applicants may hope to increase their likelihood of matching by completing more rotations, as it has been reported in the past that students were 1.5 times more likely to match at a program where they rotated [5].

In our study, 61.1% of respondents reported an average monthly cost of sub-Is of >$1600. A 2015 survey of residency applicants reported an average cost per away rotation of $958 across all specialties and an average total cost of away rotations of $2937 for orthopedic applicants [6]. A subsequent study utilizing the 2019-2020 Texas Seeking Transparency in Applying to Residency database reported a total cost to complete away rotations of $3182 for orthopedic applicants [10]. Future studies should collect cost metrics for sub-I rotations completed at home institutions and outside institutions separately. This would allow a more accurate estimation of overall cost, permit more direct comparison to prior studies, and highlight differences in sub-I practices for applicants with and without home orthopedic programs.

Thirty percent (57/190) of respondents in this study stated that their time on away rotations was limited secondary to cost. Applicants with less financial resources may therefore be at a competitive disadvantage when applying to orthopedic residency. Despite this observation, respondents found scholarships of lesser importance when determining where to apply (mean importance {S}=2.1 out of 5 {1.3}). For applicants whose time on rotations was limited by cost, the reported importance of scholarships was only marginally greater (mean {SD}=2.4 {1.4}). Future studies should seek to identify the characteristics of applicants for whom cost was a limiting factor and might investigate methods of improving the availability and distribution of cost-alleviating efforts, such as scholarships.

Applicant preferences

Given the investment required, programs should strive to provide applicant-focused away rotation experiences. To this effect, we sought to determine factors that applicants deemed most useful when determining the rank order list. Overall, applicants found residents most influential when determining rank order. This is in keeping with earlier studies citing interactions with residents as the highest-value activity on away rotations [7]. Programs may consider incorporating applicant-resident extracurricular activities into their away rotation experience.

To our knowledge, this is the first study to report factors considered by applicants when determining where to apply for sub-Is. Overall, the most important consideration was geographic location. Program reputation, word of mouth, perceived competitive fit, prior fellowship matches, and a history of matching applicants similar to yourself were of similar and relatively high importance as well. These findings largely parallel the 2023 NRMP Applicant Survey, which found perceived goodness of fit, geographic location, program reputation, and preparation for fellowship as four of the six most frequently cited factors by orthopedic applicants when determining where to apply for residency [8]. Interestingly, factors that programs might utilize for recruitment purposes, such as social media, the program website, and scholarship opportunities, were some of the least important considerations in this study.

When determining where to apply for away rotations, women found program diversity much more important than men. In 2020, women comprised 16% of orthopedic residents, an increase of only 1.2% since 2012 [11]. Given the continued gender gap in orthopedics, women pursuing orthopedic residency may prefer to match at a program where they can better identify with the resident cohort [12,13].

Osteopathic applicants found the program’s history of matching applicants similar to themselves more important than allopathic applicants. While all applicants may apply to any program since the Accreditation Council for Graduate Medical Education (ACGME) single accreditation system transition in 2020, it appears that many programs continue to demonstrate a preference for either allopathic or osteopathic applicants. Given their lower match rate, osteopathic applicants may choose to rotate at more “historically osteopathic” programs to increase their likelihood of matching [9,14].

Limitations

As a pilot study of applicants to two affiliated orthopedic residencies in the Philadelphia metropolitan area, our findings should be interpreted cautiously. Our survey was administered to 936 out of 1492 (62.7%) orthopedic applicants in the 2024 match cycle [9], and our response rate (20.3%) was lower than most health science education studies, placing our study at risk for nonresponse bias [15]. Based on NRMP data from the 2024 match, our sample correctly represents the gender distribution of orthopedic applicants but over-represents osteopathic applicants by approximately 8% [9]. We identified notable differences in the number of away rotations between allopathic and osteopathic applicants, suggesting that our reported average number of sub-I rotations may be inflated by the over-representation of osteopathic applicants. There may also be regional differences in away rotation cost, practices, and preferences, which our survey may fail to account for. A 2021 study found that the cost of orthopedic away rotations for students in the Northeast ($3297) was greater than for students in the Midwest ($2413) but was not different than costs for students in the South ($3343) or West ($3831) [10].

Despite efforts to ameliorate this, it is possible that applicants who rotated or interviewed with our programs may have felt more compelled to complete the survey, raising a concern for potential response bias. Our survey was anonymous and administered after the rank list certification deadline to make it clear that participation had no bearing on our rank lists. The survey also closed prior to match week. The limited timeframe in which the survey was available likely contributed to a low response rate, but we felt that this timing was important to minimize confirmation bias. If the survey was extended beyond match week, applicants might respond in keeping with the ideals of the program at which they matched or choose not to respond if they did not match.

Additional research is needed to better understand the role of sub-Is in the current application process. Match status was not collected in our study. Going forward, it would be useful to compare rotation practices between those who did and did not successfully match. While prior studies have demonstrated the value of away rotations in matching into orthopedic residency, it is not known whether completing more away rotations is associated with an increased likelihood of a successful match [3-5]. Additionally, a more complete understanding of how rotation practices change across different applicant characteristics would be useful. Such characteristics might include demographic factors (race/ethnicity, mean household income, and gender with the consideration of intersectionality), geographic location, the perceived competitiveness of one’s application (e.g., research experience and USMLE Step 2/COMLEX Level 2 scores), reapplicant status, and medical school characteristics (e.g., pass/fail versus graded courses and access to a home orthopedic program versus perceived support from home orthopedic program).

## Conclusions

In conclusion, this pilot study found that a majority of respondents completed multiple sub-internships (inclusive of home rotations), with osteopathic applicants generally completing more rotations than allopathic applicants. Cost emerged as a limiting factor for a portion of applicants. We also report applicant preferences when selecting away rotations and determining their rank order. Current orthopedic applicants may be completing more sub-Is at greater cost, but larger, nationally representative samples are needed to confirm these findings and to determine whether sub-Is represent a barrier to entry in orthopedics.

## Appendices

Table 4 shows the survey. 

**Table 4:** Survey.

Survey	
What is your gender?	Man
	Woman
	Others
	If others, please specify: ____________
What type of medical school do you attend?	US allopathic medical school
	US osteopathic medical school
	International medical school
How many orthopedic audition/sub-I rotations did you complete?	1
	2
	3
	4
	5
	>5
What was the average duration of the orthopedic audition/sub-I rotations that you completed?	<2 weeks
	2 weeks
	4 weeks
	>4 weeks
How long did you need to rotate with a given orthopedic surgery program to form an opinion/make a decision about rank order?	<1 week
	1-2 weeks
	2-4 weeks
	>4 weeks
While on audition/sub-I rotation, how influential were interactions with the following individuals in forming an opinion/making a decision about rank order? Please rank each individual(s) on a scale from 1 to 5 with 1 being not influential and 5 being extremely influential	
Residents	1-5
Program director	1-5
Other surgeons on faculty	1-5
Program coordinator	1-5
Ancillary/support staff	1-5
While on audition/sub-I rotations, how useful was time spent in the following settings in forming an opinion/making a decision about rank order? Please rank each setting on a scale from 1 to 5 with 1 being not useful and 5 being extremely useful	
Clinic	1-5
Operating room	1-5
Consult service/floor coverage	1-5
Conference/didactics	1-5
Interactions with residents	1-5
Did your audition rotations offer housing options for rotating students?	No
	Yes
	Sometimes
Were housing conditions suitable?	Yes
	No
Select all that apply in regard to your housing	Not clean
	Dangerous location
	Felt unsafe
	Too expensive
	Others
	Not applicable
	If others, please specify: ____________
How did you house yourself?	Rotated locally
	Residents renting a room
	Lived with friends
	Hotel
	Airbnb/rotating room
	Others
	If others, please specify: ____________
What was the average cost of housing over a one-week period while on audition/sub-I rotations?	$0 to free
	$1-$100
	$101-200
	$201-300
	$301-400
	$401-500
	>$500
	Unsure
What was the average overall cost to complete an audition/sub-I rotation over a one-week period, including housing, transportation, and food?	$0-100
	$101-200
	$201-300
	$301-400
	$401-500
	$501-1000
	>$1000
	Unsure
Was your time on audition/sub-I rotations limited secondary to cost?	Yes
	No
Please indicate how important the following factors were in determining where to apply for orthopedic audition/sub-I rotations. Please rank each factor on a scale from 1 to 5 with 1 being not important and 5 being extremely important	
Program reputation	1-5
Geographic location	1-5
Urban versus suburban versus rural	1-5
Program history of matching applicants like you	1-5
Word of mouth	1-5
Social media presence	1-5
Program website	1-5
Diversity of program	1-5
Competitiveness of your application in comparison to competitiveness of the program	1-5
Prior fellowship matches	1-5
Scholarship opportunities for rotators	1-5
Any other factors?	Yes
	No
	If yes, please specify: ____________
	If yes, please rank other factors: 1-5

Table 5 shows other factors not listed in the survey that impacted applicants’ decisions regarding where to apply.

**Table 5:** Other Factors Considered by Applicants When Determining Where to Apply for Away Rotations

Other factors	N (%)
Research	3 (1.58%)
Research and well-known faculty	1 (0.53%)
Family location/friends in the area	1 (0.53%)
Operating autonomy and resident lifestyle	1 (0.53%)
Third-year orthopedic surgery rotation with program	1 (0.53%)
Subspecialty strength	1 (0.53%)
Understanding of students’ route to orthopedic surgery	1 (0.53%)
Pediatric program	1 (0.53%)
Visa sponsorship	1 (0.53%)
Number of women	2 (1.05%)
Ties to program	2 (1.05%)
Historical osteopathic program	2 (1.05%)
Alumni	1 (0.53%)
Program culture/structure	2 (1.05%)
What type of call is covered	1 (0.53%)
Reputation of matching	1 (0.53%)
